# Comparison of Ocular Wavefront in Seated and Supine Positions Using a Hand-Held Hartmann–Shack Aberrometer

**DOI:** 10.3390/jcm14186688

**Published:** 2025-09-22

**Authors:** Noh Eun Kwon, Nicolas Brown, Jong Hwa Jun, Seung Pil Bang

**Affiliations:** 1 Department of Ophthalmology, Keimyung University School of Medicine, Dongsan Medical Center, Daegu 42601, Republic of Korea; julie9810200@gmail.com (N.E.K.); junjonghwa@gmail.com (J.H.J.); 2 Ovitz, Rochester, NY 14604, USA

**Keywords:** wavefront aberration, Hartmann–Shack aberrometer, postural change, supine position, higher-order aberrations

## Abstract

**Background/Objectives**: Intraoperative aberrometry has gained clinical relevance in correcting aberrations during cataract and corneal refractive surgeries. As wavefront aberrations are typically measured with patients seated, while surgeries are performed supine, this study aimed to compare ocular aberrations between seated and supine positions, using a hand-held Hartmann–Shack aberrometer. **Methods**: Total ocular wavefront aberrations were measured five times consecutively from cyclopledged eyes for a 6 mm pupil, under three conditions: (1) seated with a holder-fixed mode (reference), (2) seated with a hand-held mode, and (3) supine with a hand-held mode. Condition 2 was included to assess potential measurement errors from the hand-held mode. Repeatability was assessed using the standard deviation of repeated measurements (Srm) and the intraclass correlation coefficient (ICC). Differences among the three conditions were analyzed to evaluate the effects of positional change and hand-held stability on ocular wavefront aberration measurements. **Results**: Eighteen healthy subjects (36 eyes) were enrolled. The Srms for the sphere were 0.37, 0.38, and 0.40 diopters (D); and for the cylinder, 0.10, 0.10, and 0.11 D, with no significant differences across conditions. ICC values exceeded 0.9 for both lower-order aberrations (LOAs) and higher-order aberrations (HOAs), indicating excellent repeatability. The mean root mean square HOAs (HOA_RMS) were 0.48, 0.49, and 0.45 µm, with no statistically significant differences by position (*p* = 0.913) or measurement mode (*p* = 0.966). **Conclusions**: The hand-held Hartmann–Shack aberrometer demonstrated satisfactory repeatability for LOAs and HOAs. Supine measurements did not differ from seated, supporting the feasibility of extending preoperative results to intraoperative aberrometry, regardless of positional differences.

## 1. Introduction

The development of wavefront aberrometry has enhanced the evaluation of the human optical system by providing measurements of higher-order aberrations (HOAs). Gradually, wavefront aberrometry has also extended to surgical applications, such as wavefront-guided refractive surgery [1,2] and intraoperative applications during cataract surgery. A notable example of the latter is the Optiwave Refractive Analysis (ORA), which provides real-time refractive analysis during surgeries [3,4]. This system employs the Talbot-Moiré interferometry method and uses Fast Fourier Transform combined with a two-peaks algorithm to convert spatial domain data into frequency domain. In this domain, myopic refractive errors manifest as a counterclockwise rotation, while hyperopic errors appear as a clockwise rotation [3,5]. However, the system is limited to providing basic refractive information—namely, sphere, cylinder, and axis—and does not offer measurements of HOAs.

Several studies reported that ORA aids favorable outcomes in cataract surgery by providing intraoperative, real-time refractive measurements [6,7,8]. These measurements allow for the selection of intraocular lenses (IOLs) that minimize postoperative refractive error and assist accurate intraoperative alignment of toric IOLs [9,10,11]. Recent reviews indicated that ORA could serve as a valuable adjunct for challenging cases such as eyes with prior hyperopic [12] or myopic corneal ablations [13,14]. In addition, ORA has been known to provide effective complementary support for performing limbal relaxing incisions [15,16].

Intraoperative aberrometry is typically performed with the patients in a supine position, whereas preoperative wavefront measurements in clinical settings are usually acquired in a seated posture. Several studies have reported that positional changes can induce subtle shifts in refraction, particularly affecting the axis of astigmatism due to ocular cyclotorsion [17,18,19,20,21,22,23]. One study also revealed that corneal aberrations—including total HOAs and spherical-like aberration—increase when patients are in the supine position [24].

Despite these findings, limited data exist on whether wavefront aberrations, including HOAs, are influenced by changes in body posture. Therefore, this study aims to investigate and compare ocular wavefront aberrations between seated and supine positions by using a hand-held Hartmann–Shack aberrometer. The Ovitz xwave (Ovitz Cor-poration, Rochester, NY, USA) is a dual-mode Hartmann–Shack aberrometer that can be used in both the holder-fixed and hand-held modes. Given the device’s capacity to deliver detailed and comprehensive wavefront data—and its previously validated precision and repeatability [25]—the Ovitz xwave was adopted in this study to analyze posture-related changes in both low-order aberrations (LOAs) and HOAs.

## 2. Materials and Methods

### 2.1. Ethical Considerations

This cross-sectional experimental study was conducted at Dongsan Hospital, Keimyung University School of Medicine, Daegu, South Korea. Ethical approval was granted by the institutional review board of Keimyung University Dongsan Hospital (2025-07-035), and the study adhered to the tenets of the Declaration of Helsinki. Written informed consent was obtained from all participants after a thorough explanation of the study.

### 2.2. Study Design

A total of 36 eyes from 18 participants were included. All subjects were free of ocular abnormalities except for refractive errors. Prior to measurements, a complete ophthalmic evaluation was performed, including a review of medical history, visual acuity assessment, slit-lamp examination, and fundus examination. Exclusion criteria included: any anterior or posterior segment abnormalities (e.g., dry eye, corneal/vitreous opacities, diagnosed ocular diseases, history of ocular trauma or surgery other than corneal refractive surgeries, use of contact lenses or topical ophthalmic agents, and BCVA worse than 32/20. Best corrected visual acuity (BCVA) was at least 32/20. No participants wore contact lenses or used any eye drops during the study period.

### 2.3. Wavefront Aberration Measurements

**Figure 1:**
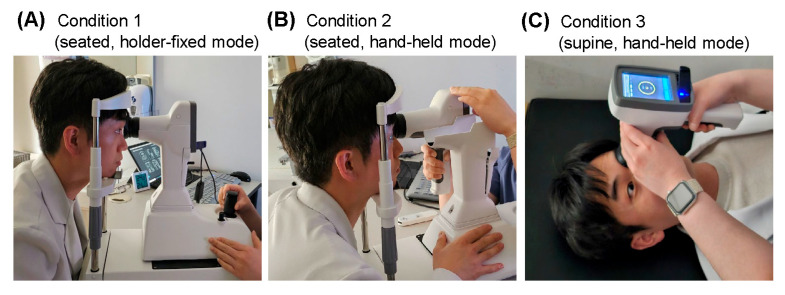
Five consecutive measurements of wavefront aberration were performed in three different conditions. (**A**) Condition 1: seated position with wavefront aberrometry in holder-fixed mode. (**B**) Condition 2: seated position with wavefront aberrometry in hand-held mode. (**C**) Condition 3: supine position with wavefont aberrometry in hand-held mode.

Total ocular wavefront aberrations were assessed using the Ovitz xwave Hartmann–Shack aberrometer. Cycloplegia was induced using a drop of 0.5% tropicamide combined with 0.5% phenylephrine in both eyes, and measurements were performed one hour later after confirming pupil dilation of at least 6 mm. Each eye underwent five consecutive measurements under three different conditions: a seated position using the holder-fixed mode (Condition 1), a seated position using the hand-held mode (Condition 2), and a supine position using the hand-held mode, immediately after reclining (Condition 3) (Figure 1). A 10 min interval was offered between each condition. The inclusion of the seated hand-held mode allowed for assessment of potential variability induced by the hand-held mode.

All measurements were performed by a single examiner. During acquisition, minimal adjustments were made between scans to reduce bias. The room was kept dark, and participants were instructed to blink completely before each scan and fixate on a central red spot emitted from the device. As an exploratory addition, Condition 4 involved five repeated measurements in the right lateral decubitus position, using the hand-held mode to assess the influence of lateral posture.

Individual Zernike coefficients from the 2nd to 6th order were analyzed for a 6 mm pupil. Total HOAs were calculated as root mean square of HOAs (HOA_RMS) from 3rd to 6th order Zernike coefficients. Wavefront aberrations were expressed in micrometers (μm), and refractive errors in diopters (D).

### 2.4. Statistical Analysis

Statistical analysis was performed using SPSS software (version 20.0; IBM Co., Armonk, NY, USA). Data were presented as mean ± standard deviation. For pairwise comparisons (e.g., seated holder-fixed vs. hand-held, seated vs. supine), Student’s *t*-tests were used. Depending on the distribution assessed via the Shapiro–Wilk normality test, either the *t*-test or the Wilcoxon signed-rank tests were employed for subgroup analysis. Bonferroni correction was used for adjusting multiple comparisons. Intraobserver repeatability was evaluated using the standard deviation of repeated measurements (Srm) and the intraclass correlation coefficient (ICC). The Srm reflects measurement variability, while the ICC assesses consistency across repeated measures. ICC values were interpreted as follows: >0.9 (excellent), 0.75–0.9 (good), 0.5–0.75 (moderate), and <0.5 (poor repeatability).

## 3. Results

Eighteen participants (36 eyes) were enrolled in the study, with a mean age of 33.3 ± 6.9 years (ranging from 23 to 51 years). Fourteen participants were female and four were male. Seven participants had previously undergone corneal refractive surgery: three had small-incision lenticule extraction (SMILE), one had laser-assisted in situ keratomileusis (LASIK), and three had laser-assisted subepithelial keratectomy (LASEK). All included eyes were free of ocular pathology.

The mean values and standard deviation of refractive errors and ocular wavefront aberrations under three different conditions are presented in Table 1. Sphere and cylinder magnitudes are presented in diopters (D), astigmatic axis in degrees (°), and Zernike coefficients in micrometers (μm). Zernike coefficients of fifth order or higher were excluded from the tables below, as most values were below the third decimal place and considered to lack clinical relevance.

**Figure 2:**
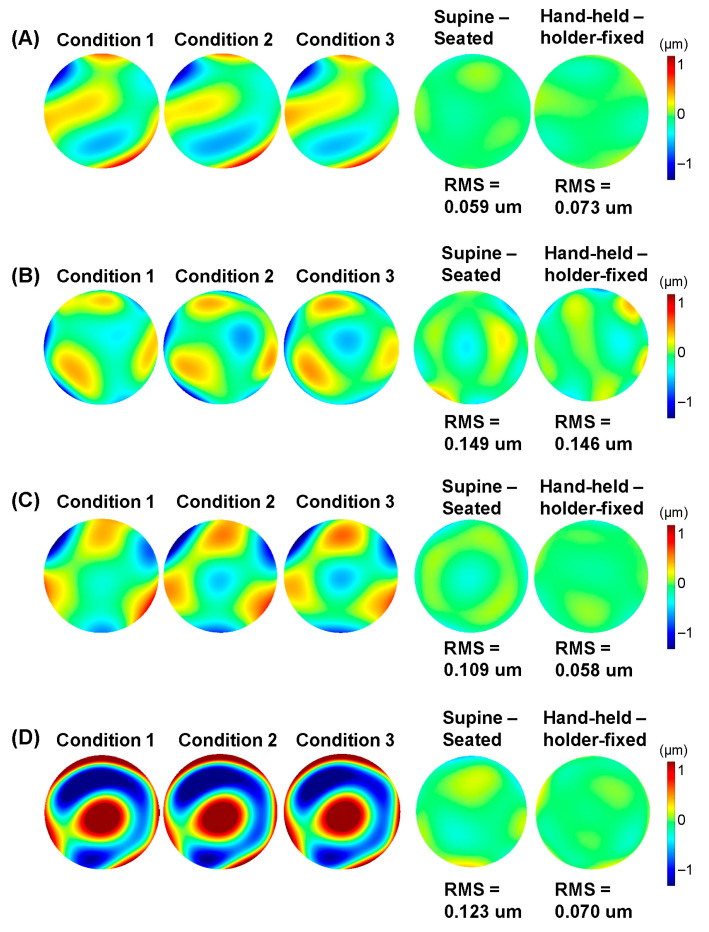
HOA wavefront maps under three conditions and their differences with RMS values. Four representative cases are shown under three different conditions ((1) seated position with holder-fixed mode, (2) seated position with hand-held mode, (3) supine position with hand-held mode) and their differences (supine–seated, hand-held–holder-fixed): (**A**) a 37 year old emmetropic female with no history of ocular surgery, (**B**) a 37 year old male with high myopia (SE < −10 D) and no history of ocular surgery, (**C**) a 25 year old female who underwent SMILE 4 years ago, and (**D**) a 41 year old female who underwent LASIK 25 years ago. No significant changes in HOAs were observed.

The pairwise comparisons across the three conditions are shown in Table 1, assessing the impact of posture and potential hand-held instability. No significant differences were observed in refractive or wavefront aberration measurements between any of the conditions (*p* > 0.05). Specifically, the mean HOA_RMS was 0.48, 0.49, and 0.45 µm for Conditions 1, 2, and 3, respectively, with no significant differences due to posture (*p* = 0.913) or measurement mode (*p* = 0.966). Representative HOA wavefront maps from four participants are shown in Figure 2.

Table 2 summarizes repeatability measures across the three conditions. The standard deviations of repeated measurements (Srm) for the sphere were 0.37, 0.38, and 0.40 D, and for the cylinder, 0.10, 0.10, and 0.11 D, for Condition 1, 2, and 3, respectively, suggesting consistent measurement repeatability. The astigmatic axis showed the highest variability, with Srm values of 5.00°, 5.64°, and 6.91° across the three conditions, respectively. For 3rd- and 4th-order Zernike terms, the largest Srm values were observed in spherical aberration (SA) (0.055 μm) for Condition 1, vertical coma (0.057 μm) for Condition 2, and SA (0.045 μm) for Condition 3. The ICCs indicated excellent repeatability (>0.9) for both LOAs and HOAs in all conditions.

A subgroup analysis was performed on eyes with cylinder values ≥ 0.75 D (n = 18), focusing on astigmatic axis variability. This threshold was selected based on previous findings indicating that astigmatism of 0.75 D or greater leads to clinically significant degradation in visual acuity [26,27]. In this group, Srm for axis was reduced to 1.99°, 2.15°, 2.28° across the three conditions, and ICCs were consistently high (0.99), as shown in Table 3. Astigmatic axis comparisons between holder-fixed vs. hand-held mode in the seated position and seated vs. supine position in the hand-held mode showed no significant differences (*p* > 0.05, Table 4). Postural changes from seated to supine showed broad distribution of astigmatic axis shifts: fourteen eyes within 5°, three eyes between 5° and 10°, and one eye greater than 10°, with a maximum of 11.2°. The overall mean cyclotorsion from the seated to supine position was 3.95 ± 2.67°, with a greater mean in right eyes (5.28 ± 2.87°) than in left eyes (2.62 ± 1.58°). Incyclotorsion was observed in eleven eyes (61%, seven right eyes and four left eyes), and excyclotorsion in seven eyes (38%, two right eyes and five left eyes) (Table 5).

A supplementary subgroup analysis was conducted for the right decubitus position (Condition 4) in eight eyes, compared with the seated and supine positions using the hand-held mode (Condition 2 and 3), including repeatability test (Supplementary Tables S1 and S2). The astigmatic axis in the right decubitus position (–1.83 ± 25.36°) differed significantly from both the seated (23.10 ± 40.33°, Condition 2; *p* = 0.0017) and supine positions (16.78 ± 35.21°, Condition 3; *p* = 0.0092). Measurement repeatability was lower in the right decubitus position, with an ICC of 0.769. Among HOAs, the maximum Srm was for vertical coma (0.062 µm), followed by vertical trefoil (0.044 µm) in the right decubitus position.

## 4. Discussion

In the current study, we investigated whether postural changes affect total ocular aberration patterns and assessed the repeatability of both LOAs and HOAs using the hand-held Hartmann–Shack aberrometer (the Ovitz xwave). Measurements were repeated five times in each condition and the wavefront aberrometer demonstrated excellent repeatability across all conditions. No significant differences in aberrations were observed due to changes in posture or measurement mode, supporting the feasibility of extending its application to intraoperative wavefront assessments during surgery.

A sufficient level of repeatability is essential to ensure that any observed posture-related aberration changes are not attributable to instrumental noise or measurement inconsistency. To date, limited published data exists regarding the repeatability or precision of the Ovitz xwave. Han et al. investigated the accuracy of this hand-held Hartmann–Shack wavefront aberrometer by comparing its measurements with those from an autokerato-refractometer and cycloplegic retinoscopy [28]. Although repeatability was not assessed, the device demonstrated reliable spherical equivalents measurements.

Previous studies have evaluated the repeatability of other Hartmann–Shack aberrometers, often using the ICC as a metric. Otero et al. and McBee et al. reported good to excellent repeatability (ICC > 0.75 for LOAs and >0.9 for most HOAs) using the VAO aberrometer [29,30]. López-Miguel et al. and Xu et al. reported similar findings with the Topcon KR-1W (ICC > 0.85) [31,32]. Visser et al. compared four different wavefront aberrometers and found that the Irx3, a Hartmann–Shack aberrometer, exhibited the smallest Srm, followed by Keratron, a hybrid Hartmann–Shack/Placido device, and other devices using ray-tracing or automated retinoscopy [33].

For refractive errors, López-Miguel et al. found intrasubject standard deviations of 0.20 D for sphere and 0.40 D for cylinder with the Topcon KR-1W [31]. Otereo et al. and McBee et al. reported within-subject standard deviations of sphere and cylinder as 0.17D, 0.15 D, and 0.18 D and 0.13 D, 0.18 D, and 0.27 D for young healthy eyes, pseudophakic eyes without refractive surgery, and pseudophakic eyes with refractive surgery, respectively, using the VAO aberrometer [29,30]. These findings indicated that spherical variability under 0.25 D is clinically acceptable, while caution is warranted with cylinder measurements near the 0.75 D threshold for clinically meaningful change [29,34,35,36]. For HOAs, Srm values of 0.041 μm for primary coma and 0.033 μm for primary SA have been reported with a 6 mm pupil [31].

In the present study, all ICCs exceed 0.9, demonstrating excellent repeatability. Cylinder Srm for cylinder (0.10–0.11D) was well within the acceptable range, while sphere Srm (0.37–0.40 D) remained below the minimal clinically significant change of 0.50 D [36]. The highest Srm were 0.057 μm for vertical coma and 0.055 μm for SA. These values fall below previously reported blur limits for 5 mm pupil—±0.15 μm for coma and ±0.16 μm for SA [37]—and remain acceptable even with our 6 mm pupil analysis. Thus, the repeatability of both LOAs and HOAs in this study can be considered to be satisfactory.

Studies exploring the effect of posture on ocular aberrations are limited. Kawamorita et al. reported that among corneal aberrations, total HOAs and spherical-like aberrations increased in the supine position, despite no significant changes in the vertical axis [24]. They hypothesized that increased IOP, though not directly measured, and gravitational effects could be contributing factors. In contrast, the current study found no significant changes in either LOAs or HOAs between the supine and seated positions, nor between the hand-held and holder-fixed modes (*p* > 0.05). This discrepancy may be attributed to the difference between corneal and total ocular aberrations, as the latter encompasses contributions from intraocular structures, including the lens and zonules. It is plausible that posture-induced changes in the cornea were offset by compensatory changes within intraocular components, resulting in stable overall aberration profiles. Further studies would be necessary to test this hypothesis.

Cyclotorsion and axis shift due to postural changes are important considerations in cataract and refractive surgeries. Various techniques—such as a Jackson cross cylinder [38], Maddox double-rod measurements [39], a keratometer [40], and an image-guided registration system [17,18,19,20,21,22,23]—have been utilized to quantify cyclotorsion. Kim et al. and others have reported mean absolute cyclotorsion of 2.6–3.2 ° of in young subjects [20,21,22,23], while older patients undergoing toric IOL implantation experienced mean shifts of 5.84 ± 3.25° [17,18,19]. Terauchi et al. reported maximum cyclotorsion of 12° [17]. Our subgroup analysis in eyes with cylinder ≥0.75 D revealed a mean axis shift of 3.95 ± 2.67° and a maximum of 11.2°, consistent with previous reports with high repeatability (ICC = 0.99). Regarding directionality, while Febbraro et al. and others observed predominance of excyclotorsion (60%) [20,21,23], Hummel et al. and Srujana et al. reported more frequent incyclotorsion (67.4%) [18,19], which aligns with our results (61% incyclotorsion).

Postural changes, particularly from seated to supine, are known to increase intraocular pressure (IOP) [41,42,43,44,45,46]. Lam et al. reported significantly increased IOP and mild vertical axis shifts without significant central corneal curvature change via conventional keratometry [41]. In our study, IOP was not measured, to avoid corneal deformation during examination. Nevertheless, posture-related changes in IOP or cyclotorsion did not significantly influence total ocular wavefront aberrations. This may be attributed to the fact that measurements were taken immediately after assuming the supine position. However, as changes in ocular aberrations over longer durations in the supine posture cannot be ruled out, further studies are warranted to investigate this possibility.

An additional subgroup analysis including the right lateral decubitus position demonstrated significant astigmatic axis difference compared to the seated and supine positions (*p* < 0.05), with relatively low repeatability (ICC = 0.769). While overall, HOA_RMS remained stable, vertical coma and trefoil showed greater Srm variability in the decubitus position. Several mechanisms could explain these findings. First, the vestibulo-ocular reflex effect may be more pronounced in the lateral decubitus position, resulting in greater cyclotorsion [47,48]. Second, changes in gravitational vectors may induce subtle shifts in intraocular fluid, zonular tension, and extraocular muscle tone [49,50,51]. Third, IOP elevation in the dependent eye during the lateral decubitus position may contribute further variability [43,52,53]. Lastly, minor head tilts or misalignments during the lateral decubitus position might have introduced additional variability [54,55].

This study has several limitations. Intraobserver repeatability was not assessed. In addition, the absence of objective verification of full cycloplegia may have contributed to variability in spherical errors. The sample was relatively small and limited to young, healthy participants. Given the influence of age-related changes in tear film stability [56,57], ocular anatomy, and wavefront aberrations [58,59], further research should include broader and older populations. Lastly, as our study focused exclusively on total ocular aberrations, additional studies are needed to evaluate the individual contributions and interrelationships of corneal, internal, and total ocular aberrations in relation to postural changes.

In conclusion, the Ovitz xwave, a hand-held Hartmann–Shack aberrometer, demonstrated satisfactory repeatability for both LOAs and HOAs. Neither postural changes (seated vs. supine) nor hand-held measurement instability significantly influenced ocular aberrations. These results support the feasibility of hand-held aberrometry in patients who are unable to maintain the upright position, including those with postural disabilities or ambulation difficulties. As refractive surgery becomes increasingly prevalent [60], precise assessment of astigmatism and IOL targeting—especially in post-refractive eyes—is critical [61]. This study supports the potential extension of Hartmann–Shack aberrometry to intraoperative settings in modern cataract surgery, demonstrating reliable performance regardless of patient positioning.

## Figures and Tables

**Table 1:** Values of refractive power and ocular wavefront aberration and comparisons among three conditions, positional change, and hand-held stability.

Parameter	Mean ± SD			*p*-Values	
	Condition 1	Condition 2	Condition 3	Condition 2 vs. Condition 3	Condition 1 vs. Condition 2
Sph (D)	−3.10 ± 2.74	−3.36 ± 2.79	−3.40 ± 2.85	1	1
Cyl (D)	−0.99 ± 0.76	−0.98 ± 0.73	−0.96 ± 0.84	1	1
Axis (°)	23.16 ± 49.32	26.79 ± 42.77	22.82 ± 40.35	0.210	0.326
Z(3, −3), V. Trefoil	−0.072 ± 0.18	−0.077 ± 0.19	−0.055 ± 0.17	0.724	1
Z(3, −1), V. Coma	0.031 ± 0.32	0.024 ± 0.31	0.048 ± 0.29	0.382	0.686
Z(3, 1), H. Coma	0.045 ± 0.16	0.063 ± 0.18	0.057 ± 0.17	0.514	0.566
Z(3, 3), H. Trefoil	−0.025 ± 0.11	−0.036 ± 0.12	−0.038 ± 0.12	0.566	0.292
Z(4, −4), Ob. Quadrafoil	0.001 ± 0.05	−0.001 ± 0.05	0.002 ± 0.05	0.928	0.550
Z(4, −2), Ob. 2nd Astig	0.003 ± 0.05	0.003 ± 0.05	0.006 ± 0.04	0.764	1
Z(4, 0), Spherical	0.13 ± 0.19	0.11 ± 0.21	0.06 ± 0.21	0.748	0.466
Z(4, 2), V. 2nd Astig	−0.033 ± 0.11	−0.024 ± 0.12	−0.012 ± 0.12	0.306	0.916
Z(4, 4), V. Quadrafoil	0.029 ± 0.08	0.029 ± 0.09	0.033 ± 0.09	1	1
HOA_RMS	0.45 ± 0.24	0.46 ± 0.25	0.42 ± 0.25	1	1

Condition 1 = seated position with holder-fixed mode; Condition 2 = seated position with hand-held mode; Condition 3 = supine position with hand-held mode; SD = standard deviation; V. Trefoil = Vertical Trefoil; V. Coma = Vertical Coma; H. Coma = Horizontal Coma; H. Trefoil = Horizontal Trefoil; Ob. Quadrafoil = Oblique Quadrafoil; Ob. 2nd Astig = Oblique Secondary Astigmatism; Spherical = Spherical Aberration; V. 2nd Astig = Vertical Secondary Astigmatism; V. Quadrafoil = Vertical Quadrafoil; HOA_RMS = root mean square higher-order aberration (3rd to 6th order). All values analyzed for 6 mm pupil diameter. Spherical, cylindrical powers presented in Diopters (D), axis in degrees (°), other values in micrometer (μm). *p* > 0.05; Student’s *t*-test with Bonferroni correction.

**Table 2:** Repeatability test of five consequential measurements in three different conditions.

Parameter	Srm			ICC		
	Condition 1	Condition 2	Condition 3	Condition 1	Condition 2	Condition 3
Sph (D)	0.37	0.38	0.40	0.937	0.965	0.916
Cyl (D)	0.10	0.10	0.11	0.920	0.957	0.967
Axis (º)	5.00	5.64	6.91	0.918	0.906	0.958
Z(3,−3), V. Trefoil	0.038	0.021	0.033	0.988	0.986	0.975
Z(3,−1), V. Coma	0.049	0.057	0.039	0.932	0.911	0.925
Z(3, 1), H. Coma	0.039	0.031	0.039	0.914	0.912	0.901
Z(3, 3), H. Trefoil	0.034	0.027	0.032	0.924	0.975	0.915
Z(4,−4), Ob. Quadrafoil	0.026	0.023	0.025	0.981	0.986	0.914
Z(4,−2), Ob. 2nd Astig	0.021	0.017	0.018	0.911	0.915	0.923
Z(4, 0), Spherical	0.055	0.051	0.045	0.968	0.919	0.909
Z(4, 2), V. 2nd Astig	0.030	0.026	0.025	0.932	0.922	0.933
Z(4, 4), V. Quadrafoil	0.029	0.026	0.024	0.956	0.957	0.963

Condition 1 = seated position with holder-fixed mode; Condition 2 = seated position with hand-held mode; Condition 3 = supine position with hand-held mode; Srm = Standard deviation of repeated measurements; ICC = intraclass correlation coefficient; V. Trefoil = Vertical Trefoil; V. Coma = Vertical Coma; H. Coma = Horizontal Coma; H. Trefoil = Horizontal Trefoil; Ob. Quadrafoil = Oblique Quadrafoil; Ob. 2nd Astig = Oblique Secondary Astigmatism; Spherical = Spherical Aberration; V. 2nd Astig = Vertical Secondary Astigmatism; and V. Quadrafoil = Vertical Quadrafoil.

**Table 3:** Values and repeatability test in subjects with cylinder 0.75 diopters or more.

Parameter	Condition 1	Condition 2	Condition 3
Mean ± SD (°)	0.090 ± 10.96	−1.700 ± 11.90	−3.733 ± 10.97
Srm	1.991	2.149	2.280
ICC	0.99	0.99	0.99
Axis deviation			
axis ≤ ±5°	87 (97)	89 (99)	89 (99)
±5° < axis ≤ ±10°	3 (3)	1 (1)	1 (1)
axis > ±10°	0 (0)	0 (0)	0 (0)

Condition 1 = seated position with holder-fixed mode; Condition 2 = seated position with hand-held mode; Condition 3 = supine position with hand-held mode; Srm = Standard deviation of repeated measurements; ICC = intraclass correlation coefficient; axis deviation = axis measurement − mean of each repeated measurement. Axis deviation was measured five times per eye (18 eyes, 90 measurements in total). Data are presented as number (%).

**Table 4:** Comparison of changes in astigmatic axis among three conditions, hand-held stability, and posture change in subjects with cylinder 0.75 diopters or more.

Difference in Axis (n = 18)	Conditions 1 and 2	Conditions 2 and 3
mean ± SD (°)	2.58 ± 3.05	3.95 ± 2.67
*p*-value	0.284	0.148
Δ axis		
Δ axis < ±3°	15 (83)	5 (28)
Δ axis ≤ ±5°	15 (83)	14 (78)
±5° < Δ axis ≤ ±10°	3 (17)	3 (17)
Δ axis > ±10°	0 (0)	1 (6)

Condition 1 = seated position with holder-fixed mode; Condition 2 = seated position with hand-held mode; Condition 3 = supine position with hand-held mode; mean ± SD = mean and standard deviation from the means of repeated measurement; Δ axis = difference in mean values of repeated measurements. *p* > 0.05: Wilcoxon signed-rank test with Bonferroni correction, and Δ axis is presented as number (%).

**Table 5:** Measurement of cyclotorsion and the direction of rotation in right and left eyes.

Parameter	Mean Cyclotorsion (°)	Incyclotorsion (n)	Excyclotorsion (n)
Right eye (n = 9)	5.28 ± 2.87	7 (77%)	2 (22%)
Left eye (n = 9)	2.62 ± 1.58	4 (44%)	5 (55%)
Total (n = 18)	3.95 ± 2.67	11 (61%)	7 (38%)

Mean cyclorotation is presented as mean and standard deviation. The direction of rotation is presented as number (%).

## Data Availability

The datasets analyzed or generated during the current study are available from the corresponding author on reasonable request. S.P.B. has full access to all the data in the study and takes responsibility for the integrity of the data and accuracy of the data analysis.

## Supplementary Materials

The following supporting information can be downloaded at: https://www.mdpi.com/article/10.3390/jcm14186688/s1, Table S1: Values and comparisons among postural change—seated, supine and decubitus; Table S2: Repeatability test of five consequential measurements in three different conditions.
