# Supplementary material for: Comparison of Ocular Wavefront in Seated and Supine Positions Using a Hand-Held Hartmann–Shack Aberrometer
Source: J Clin Med. 2025 Sep 22;14(18):6688. doi: 10.3390/jcm14186688 (PMC12470436; doi:10.3390/jcm14186688)
Supplement: Supplementary file 1 [file jcm-14-06688-s001.zip › jcm-3856482-supplementary.pdf]

Table S1. Values and comparisons among postural change – seated, supine and decubitus.

Parameter	Mean ± SD			P-values		
	Condition 2	Condition 3	Condition 4	2vs3	2vs4	3vs4
Sph (D)	-2.519 ± 1.61	-2.669 ± 1.42	-2.658 ± 1.20	0.665	0.671	0.339
Cyl (D)	-0.669 ± 0.38	-0.7 ± 0.43	-0.71 ± 0.44	0.197	0.907	0.913
Axis (º)	23.100 ± 40.33	16.775 ± 35.21	-1.825 ± 25.36	0.463	**0.0017***	**0.0092***
Z(3,-3), V. Trefoil	-0.040 ± 0.18	-0.039 ± 0.15	-0.002 ± 0.15	0.975	0.308	0.282
Z(3,-1), V. Coma	-0.165 ± 0.38	-0.122 ± 0.31	-0.079 ± 0.32	0.588	0.284	0.552
Z(3, 1), H. Coma	0.124 ± 0.21	0.128 ± 0.20	0.131 ± 0.18	0.807	0.807	0.752
Z(3, 3), H. Trefoil	-0.004 ± 0.13	0.010 ± 0.12	-0.023 ± 0.10	0.638	0.471	0.206
Z(4,-4), Ob. Quadrafoil	0.007 ± 0.08	-0.002 ± 0.07	0.010 ± 0.09	0.592	0.888	0.528
Z(4,-2), Ob. 2nd Astig	0.018 ± 0.07	0.004 ± 0.05	0.012 ± 0.09	0.314	0.749	0.610
Z(4, 0), Spherical	-0.100 ± 0.17	-0.122 ± 0.18	-0.098 ± 0.12	0.601	0.936	0.497
Z(4, 2), V. 2nd Astig	0.082 ± 0.20	0.085 ± 0.20	0.083 ± 0.17	0.942	0.981	0.958
Z(4, 4), V. Quadrafoil	-0.030 ± 0.16	-0.021 ± 0.16	-0.023 ± 0.13	0.821	0.851	0.952
HOA_RMS	0.583 ± 0.25	0.545 ± 0.21	0.503 ± 0.20	0.479	0.129	0.382

Condition 2 = seated position with hand-held mode ; Condition 3 = supine position with hand-held mode ; Condition 4 = right decubitus position with hand-held mode ; SD = standard deviation ; V. Trefoil = Vertical Trefoil ; V. Coma = Vertical Coma ; H. Coma = Horizontal Coma ; H. Trefoil = Horizontal Trefoil ; Ob. Quadrafoil = Oblique Quadrafoil ; Ob. 2nd Astig = Oblique Secondary Astigmatism ; Spherical = Spherical Aberration ; V. 2nd Astig = Vertical Secondary Astigmatism ; V. Quadrafoil = Vertical Quadrafoil ; HOA_RMS = root mean square higher-order aberration (3rd to 6th order)

* $p < 0.05$; Student's t-test

All values analyzed for 6-mm pupil diameter. Spherical, cylindrical powers presented in Diopters(D), axis in degrees(º), other values in micrometer(μm).

Table S2. Repeatability test of five consequential measurements in three different conditions.

Parameter	S_{rm}			ICC
	Condition 2	Condition 3	Condition 4	Condition 4
Sph (D)	0.465	0.454	0.395	0.970
Cyl (D)	0.050	0.088	0.074	0.987
Axis (º)	4.572	7.664	6.430	**0.769**
Z(3,-3), V. Trefoil	0.037	0.018	0.044	0.961
Z(3,-1), V. Coma	0.051	0.034	**0.062**	0.991
Z(3, 1), H. Coma	0.023	0.025	0.028	0.996
Z(3, 3), H. Trefoil	0.034	0.022	0.033	0.969
Z(4,-4), Ob. Quadrafoil	0.022	0.021	0.029	0.962
Z(4,-2), Ob. 2nd Astig	0.022	0.020	0.031	0.947
Z(4, 0), Spherical	0.061	0.049	0.033	0.985
Z(4, 2), V. 2nd Astig	0.029	0.024	0.026	0.996
Z(4, 4), V. Quadrafoil	0.037	0.020	0.020	0.995

Condition 2 = seated position with hand-held mode ; Condition 3 = supine position with hand-held mode ; Condition 4 = right decubitus position with hand-held mode ; S_{rm} = Standard deviation of repeated measurements ; ICC = intraclass correlation coefficient ; ; V. Trefoil = Vertical Trefoil ; V. Coma = Vertical Coma ; H. Coma = Horizontal Coma ; H. Trefoil = Horizontal Trefoil ; Ob. Quadrafoil = Oblique Quadrafoil ; Ob. 2nd Astig = Oblique Secondary Astigmatism ; Spherical = Spherical Aberration ; V. 2nd Astig = Vertical Secondary Astigmatism ; V. Quadrafoil = Vertical Quadrafoil
